# Maternal Genistein Alters Coat Color and Protects *A^vy^* Mouse Offspring from Obesity by Modifying the Fetal Epigenome

**DOI:** 10.1289/ehp.8700

**Published:** 2006-01-26

**Authors:** Dana C. Dolinoy, Jennifer R. Weidman, Robert A. Waterland, Randy L. Jirtle

**Affiliations:** 1 Department of Radiation Oncology, Duke University Medical Center, Durham, North Carolina, USA; 2 University Program in Genetics and Genomics and; 3 Integrated Toxicology Program, Duke University, Durham, North Carolina, USA; 4 Department of Pediatrics and; 5 Department of Molecular and Human Genetics, Baylor College of Medicine, Houston, Texas, USA

**Keywords:** developmental origins of adult disease, DNA methylation, epi-genetics, viable yellow agouti (*A^vy^*) mouse

## Abstract

Genistein, the major phytoestrogen in soy, is linked to diminished female reproductive performance and to cancer chemoprevention and decreased adipose deposition. Dietary genistein may also play a role in the decreased incidence of cancer in Asians compared with Westerners, as well as increased cancer incidence in Asians immigrating to the United States. Here, we report that maternal dietary genistein supplementation of mice during gestation, at levels comparable with humans consuming high-soy diets, shifted the coat color of heterozygous viable yellow agouti (*A^vy^*/*a*) offspring toward pseudoagouti. This marked phenotypic change was significantly associated with increased methylation of six cytosine–guanine sites in a retrotransposon upstream of the transcription start site of the *Agouti* gene. The extent of this DNA methylation was similar in endodermal, mesodermal, and ectodermal tissues, indicating that genistein acts during early embryonic development. Moreover, this genistein-induced hypermethylation persisted into adulthood, decreasing ectopic *Agouti* expression and protecting offspring from obesity. Thus, we provide the first evidence that *in utero* dietary genistein affects gene expression and alters susceptibility to obesity in adulthood by permanently altering the epigenome.

Developmental plasticity occurs when environmental influences affect cellular pathways during gestation, enabling a single genotype to produce a broad range of adult phenotypes (Bateson et al. 2004). Specifically, the developmental origins hypothesis postulates that nutrition and other environmental factors during prenatal and early postnatal development influence developmental plasticity and alter susceptibility to adult cardiovascular disease, type 2 diabetes, and obesity (Barker 1997, 2004). Moreover, persistent epigenetic adaptations that occur early in development in response to maternal nutrition and the environment are associated with increased susceptibility to cancer and other adult-onset chronic diseases (Cooney et al. 2002; Li et al. 2003; Waterland and Jirtle 2004).

Methylation of cytosines in cytosine–guanine (CpG) dinucleotides represents a critical epigenetic DNA modification affecting gene expression and cellular function (Bird 2002). Transposable elements, the promoter regions of housekeeping genes, and *cis*-acting regulatory elements of imprinted genes are three key epigenetic susceptibility targets containing CpG sites that are normally methylated, unmethylated, and differentially methylated, respectively. Therefore, environmental factors that affect DNA methylation patterning during development can potentially influence adult phenotype via alterations in CpG methylation at epigenetically labile regions in the genome.

The epigenome is likely to be most vulnerable to environmental factors during embryogenesis because the DNA synthetic rate is high, and the elaborate DNA methylation patterning required for normal tissue development is established during this period. Therefore, when evaluating the effects of environmental influences on the epigenome, not only the dose but also the developmental timing must be considered. For example, dietary methyl donor intake in adulthood is not associated with risk of breast cancer among African-American women (Zhu et al. 2003). Nevertheless, it remains possible that epigenetic modifications caused by the nutritional environment of the embryo, fetus, and neonate are involved in the etiology of this adult disease.

Isoflavones represent a class of phyto-estrogens present in soy and soy products that are active in multiple biologic systems, including estrogen-receptor– and non–estrogen-receptor–mediated signaling pathways (Lamartiniere et al. 2002; Valachovicova et al. 2004). Genistein, the major isoflavone in soy, exhibits mixed estrogen agonist and antagonist properties (Price and Fenwick 1985), inhibits tyrosine kinase (Akiyama et al. 1987), and scavenges free radicals (Wei et al. 1993), depending on timing, dose, and the tissue examined. A diet rich in soy, such as a typical Asian or Western vegetarian diet, contains as much as 1.4 mg genistein/kg body weight per day (Coward et al. 1993), whereas infants fed soy formula consume almost five times as much genistein (Setchell et al. 1997). Genistein is linked to reduced female reproductive health (Nagaos et al. 2001) but also to breast and prostate cancer chemoprevention (Lamartiniere et al. 2002) and decreased adipose deposition (Naaz et al. 2003). Thus, dietary genistein may help explain the difference in cancer incidence between Westerners and Asian populations with high soy intake (Lee et al. 1991; Peeters et al. 2003; Ziegler et al. 1993).

Despite a growing body of toxicologic and mechanistic literature on the effects of genis-tein and other phytoestrogens, the long-term health consequences of developmental and early exposure remain largely unknown (Badger et al. 2002). Limited evidence suggests that exposure to phytoestrogens post-natally alters the epigenome (Day et al. 2002; Lyn-Cook et al. 1995). Neonatal exposure to high doses of the phytoestrogens equol and coumestrol is correlated with hypermethyla-tion of a protooncogene in the rat pancreas (Lyn-Cook et al. 1995). More recently, a study employing methylation arrays suggests that adult dietary genistein induces gene hypermethylation in the prostate gland (Day et al. 2002). Interestingly, the effect of genis-tein exposure during gestation on DNA methylation in the offspring has not been investigated even though this is when the epigenome is most susceptible to environmentally induced dysregulation.

To determine if maternal genistein affects offspring by altering the epigenome *in utero*, we assessed coat color, DNA methylation, and body weight in genetically identical heterozygous viable yellow agouti (*A^vy^*/*a*) offspring. The results show that genistein-induced CpG hypermethylation of six CpG sites in the *A^vy^* intracisternal A particle (IAP) retrotransposon shifted stochastic coat-color distribution toward pseudoagouti, thereby decreasing the incidence of adult-onset obesity in *A^vy^*/*a* offspring. This is the first evidence that early *in utero* exposure to genistein results in decreased adult chronic disease susceptibility by producing permanent alterations in the epigenome.

## Materials and Methods

### Animals and diets.

*A^vy^* mice were obtained from Oak Ridge National Laboratory (Oak Ridge, TN) from a colony that has been maintained with sibling mating and forced heterozygosity for the *A^vy^* allele for more than 200 generations, resulting in a genetically invariant background (Waterland and Jirtle 2003). The *A^vy^* allele is passed through the paternal lineage to avoid bias associated with maternal transmission where methylation of the maternal allele is not completely reset (Rakyan et al. 2001).

Virgin *a*/*a* females, 8–10 weeks of age, were assigned to receive either phytoestrogen-free modified AIN-93G diet (diet 95092 with 7% corn oil substituted for 7% soybean oil; Harlan Teklad, Madison, WI) or modified AIN-93G diet supplemented with 250 mg/kg diet of genistein (diet 00417, Harlan Teklad). This level of genistein in the diet results in the animals being exposed to concentrations comparable with those received by humans consuming high-soy diets (Fritz et al. 2002). Harland Teklad supplied all diet ingredients except genistein (Indofine Chemical Company, Hillsborough, NJ). Diets were provided 2 weeks before mating females with *A^vy^*/*a* males and throughout pregnancy and lactation. At postnatal day 21, all offspring were weaned to stock maintenance diet (diet 5021; LabDiet, Richmond, IN). *A^vy^*/*a* offspring were weighed, digitally photographed, and rated for coat-color phenotype.

For *A^vy^*/*a* offspring, total DNA was isolated from day 21 tail clips, day 150 tail, day 150 liver, day 150 brain, and day 150 kidney using buffer ATL, proteinase K, and Rnase A (Qiagen Inc., Valencia, CA) followed by phenol-chloroform extraction and ethanol precipitation. Animals used in this study were maintained in accordance with the *Guidelines for the Care and Use of Laboratory Animals* (Institute of Laboratory Animal Resources 1996) and were treated humanely and with regard for alleviation of suffering. The study protocol was approved by the Duke University Institutional Animal Care and Use Committee.

### Coat-color phenotype classification and body weight measurement.

A single, blinded observer visually classified day 21 *A^vy^*/*a* offspring coat-color phenotype into one of five categories based on proportion of brown to yellow in the fur: yellow (< 5% brown), slightly mottled (between 5% and 50% brown), mottled (~ 50% brown), heavily mottled (between 50% and 95% brown), and pseudoagouti (> 95% brown). *A^vy^*/*a* offspring were weighed on a calibrated digital scale every 5 weeks from week 25 to week 60. Twenty-two animals were sacrificed for tissues or died before week 60 and were not included in the body weight analysis.

### Methylation assay.

Sodium bisulfite modification of DNA was performed using a protocol adapted from Gruanau et al. (2001) as previously described (Waterland and Jirtle 2003). Regions of interest were amplified from bisulfite-modulated DNA in 50-μL polymerase chain reaction (PCR) using 1.5 U Platinum *Taq* DNA polymerase (Invitrogen, Carlsbad, CA), 15 pmol primers, 1.5 mM MgCl_2_, and 10 mM dinucleotide triphosphates (94°C, 2 min; 94°C × 30 sec, 55°C × 30 sec, and 72°C × 60 sec for 40 cycles; 72°C, 9 min). We used forward primer IAPF3 (5′ ATT TTT AGG AAA AGA GAG TAA GAA GTA AG 3′) and reverse primer IAPR4 (5′ TAA TTC CTA AAA ATT TCA ACT AAT AAC TCC 3′) from Waterland and Jirtle (2003).

PCR products were resolved by electrophoresis on a 1.5% agarose gel, excised, gel extracted (GenElute; Sigma Chemical Co., St. Louis, MO), and sequenced manually (Thermo Sequenase Radiolabeled Terminator Cycle Sequencing kit; USB Corporation, Cleveland, OH) according to manufacturer’s instructions (95°C × 30 sec, 55°C × 30 sec, and 72°C × 60 sec for 35 cycles) using forward sequencing primer IAPF5 (5′ ATT ATT TTT TGA TTG TTG TAG TTT ATG G 3′). Sequencing products were resolved using polyacrylamide gel electrophoresis. Blank lanes were placed between C and T lanes to prevent signal overlap. Percentage of cells methylated at the nine CpG sites in the *A^vy^* IAP region was quantified by phosphor imaging (percentage of cells methylated = [100 × (C intensity)/(C intensity + T intensity)]. The nine CpG sites studied are located at nucleotide positions 206, 214, 220, 244, 265, 306, 319, 322, and 334 of GenBank accession number AF540972 (GenBank 2005).

### HPLC determination of SAM and SAH.

Hepatic concentrations of *S*-adenosylmethion-ine (SAM) and *S*-adenosylhomocysteine (SAH) were measured using the high-performance liquid chromatography (HPLC) method of Herbig et al. (2002). Approximately 50 mg of liver was weighed and homogenized in 500 μL 0.1 M sodium acetate (pH 5.5), and cellular proteins were precipitated by addition of 312 μL 10% perchloric acid. After centrifugation (2,000 × *g*, 10 min, 4°C), each supernatant was transferred to a clean tube, neutralized by addition of 140 μL 1 M sodium phosphate (pH 11.5), and diluted with 1 mL deionized H_2_O. Each sample was then applied to a C18 Sep-Pak cartridge (Waters Corp., Milford, MA) primed with 5 mM 1-heptane-sulfonic acid (Alfa Aesar, Ward Hill, MA) in methanol. The cartridges were then washed with 5 mL deionized H_2_O before SAM and SAH were eluted in 2 mL methanol. Fifty microliters of 3 M NaAcO was added to each eluate before drying under vacuum at ambient temperature overnight. SAM and SAH were resuspended in 250 μL H_2_O and converted to their fluorescent derivatives by the addition of 50 μL chloroacetylaldehyde (50% by weight; Sigma-Aldrich, Milwaukee, WI) and incubation at 60°C for 1 hr. Purified SAM (New England Biolabs, Beverly, MA) and SAH (Sigma-Aldrich) were quantitated spectropho-tometrically and used to generate standard curves. The standards were prepared using the same complete protocol as described for the tissue extracts. Samples and standards were loaded onto a C8 column (5 μm, 250 × 4.6 mm; Phenomenex, Torrance, CA), and solvent delivery was performed by two Shimadzu (Columbia, MD) LC-10ADVP pumps controlled by a SCL-10AVP system controller. A two-buffer system was used to separate SAM and SAH, as described by Herbig et al. (2002). SAM and SAH were detected with a Shimadzu RF-10AXL spectro-fluorometric detector (λ_ex_ = 270 nm and λ_em_ = 410 nm).

### Statistical analysis.

Diet group comparisons of the proportion of offspring in each of the five coat-color classes were performed by chi-square analysis. Average IAP CpG methylation and site-specific CpG methylation between the unsupplemented and genistein-supplemented groups were analyzed by two-tailed two-sample hypothesis testing of means with STATA software (version 8.0; StataCorp., College Station, TX). Relationships among genistein supplementation, *A^vy^* IAP methylation, and coat color were analyzed by mediational regression analysis (Baron and Kenny 1986). Pearson’s correlation coefficients and *p*-values of tissue type and age were calculated with STATA software. Body weight across coat-color phenotype was assessed by Bonferroni-corrected analysis of variance (ANOVA). Diet group comparisons of the body weight were performed by chi-square analysis.

## Results

### *DNA methylation and the* A^vy^ *model*.

**Figure 1:**
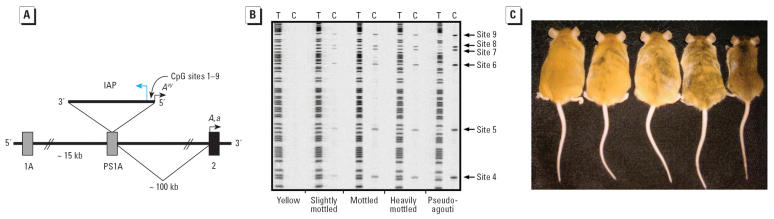
Methylation status of CpG sites within the *A^vy^* IAP in genetically identical *A^vy^*/*a* littermates. (*A*) A contraoriented IAP insertion within pseudoexon 1A (PS1A) of the murine *Agouti* gene. A cryptic promoter (short arrow labeled *A^vy^*) drives ectopic *Agouti* expression. CpG sites 1–9 are oriented in the 3′ to 5′ direction with respect to the IAP insertion, as shown. Transcription of *A* and *a* alleles initiates from a hair-cycle–specific promoter in exon 2 (short arrow labeled *A,a*). (*B*) Pseudoagouti animals exhibit the highest degree of CpG methylation at sites 4–9. Bisulfite sequencing reveals increasing intensity of the cytosine lane at CpG sites 4–9 within the *A^vy^* IAP in genetically identical *A^vy^*/*a* animals representing the five coat classes. (*C*) Genetically identical week-15 *A^vy^*/*a* mouse littermates representing the five coat-color phenotypes.

The murine *Agouti* gene encodes a paracrine signaling molecule that promotes follicular melanocytes to produce yellow phaeomelanin pigment instead of black eumelanin pigment. Transcription is normally initiated from a hair-cycle–specific promoter in exon 2 of the *agouti* (*A*) allele (Figure 1A). Transcription of the *A* allele normally occurs only in the skin where transient *A* expression in hair follicles during a specific stage of hair growth results in a subapical yellow band on each black hair, causing the brown (agouti) coat color of wild-type mice (Duhl et al. 1994).

The *A^vy^* allele resulted from the insertion of an IAP murine retrotransposon upstream of the transcription start site of the *Agouti* gene (Figure 1A) (Duhl et al. 1994; Waterland and Jirtle 2003). A cryptic promoter in the proximal end of the *A^vy^* IAP promotes constitutive ectopic *Agouti* transcription, leading to yellow fur, obesity, and tumorigenesis (Miltenberger et al. 1997; Morgan et al. 1999). CpG methylation in the *A^vy^* IAP correlates inversely with ectopic *Agouti* expression. The degree of methylation and corresponding level of ectopic *Agouti* expression vary stochastically among individual isogenic *A^vy^*/*a* mice, causing a wide variation in coat color ranging from yellow (unmethylated) to pseudoagouti (methylated) (Morgan et al. 1999) (Figure 1B,C). Increased body weight is also positively correlated to ectopic agouti expression, as seen in the week 15 isogenic *A^vy^*/*a* littermates shown in Figure 1C.

### Offspring characteristics and coat-color distribution.

**Figure 2:**
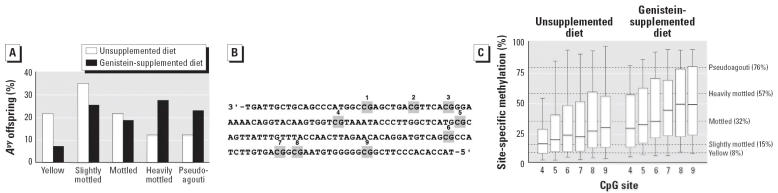
Coat-color distribution and methylation of CpG sites 4–9 of the *A^vy^* IAP in offspring whose mothers were fed unsupplemented and genistein-supplemented (250 mg genistein/kg diet) diets. (*A*) Coat-color distribution of *A^vy^*/*a* offspring born to 15 unsupplemented and 12 genistein-supplemented litters. (*B*) Genomic sequence containing nine CpG sites located between the cryptic *Agouti* promoter and the IAP promoter (blue arrow in Figure 1A) at the 5′ end of the contraoriented *A^vy^* IAP. CpG sites 1–9 are numbered and marked by gray boxes. (*C*) Box plots representing the percentage of cells methylated at sites 4–9 in unsupplemented (*n* = 52) and genistein-supplemented (*n* = 44) *A^vy^*/*a* offspring. Ends of the boxes indicate the interquartile range representing the 25th to 75th percentiles of the data; horizontal lines within each box indicate median; and dashed horizontal lines represent average percent methylation of CpG sites 4–9 according to coat-color phenotype.

The total number of offspring studied was from 15 unsupplemented litters (52 *A^vy^*/*a* offspring) and 12 genistein-supplemented litters (44 *A^vy^*/*a* offspring). Maternal genistein did not significantly influence litter size, wean weight, percent survival, or sex ratio (data not shown). Maternal genistein supplementation shifted the coat-color distribution of genetically identical day 21 *A^vy^*/*a* offspring toward the pseudoagouti phenotype (chi-square, *p* = 0.0005) (Figure 2A). Fifty percent of genistein-supplemented offspring were classified as pseudoagouti or heavily mottled, compared with 23% of unsupple-mented offspring. Furthermore, only 7% of genistein-supplemented offspring were categorized at yellow, compared with > 21% of the unsupplemented offspring.

### Genistein supplementation and DNA methylation.

Bisulfite sequencing methylation analysis (Grunau et al. 2001) of CpG sites in the cryptic promoter region of the *A^vy^* IAP (Figure 1A,1B, Figure 2B) showed a statistically increased average percentage of cells methylated in genistein-supplemented offspring (*n* = 44) relative to that in unsupple-mented offspring (*n* = 52) (two-tailed *t*-test, *p* = 0.025). Analysis of site-specific methylation at nine individual CpG sites revealed significantly different methylation between the unsupplemented and genistein-supplemented diet groups at sites 4–9 (two-tailed *t*-test, *p* = 0.004, 0.02, 0.04, 0.03, 0.05, and 0.02, respectively; Figure 2B,C). Moreover, the statistical significance of site 4 is an order of magnitude greater than that for sites 5–9.

**Figure 3:**
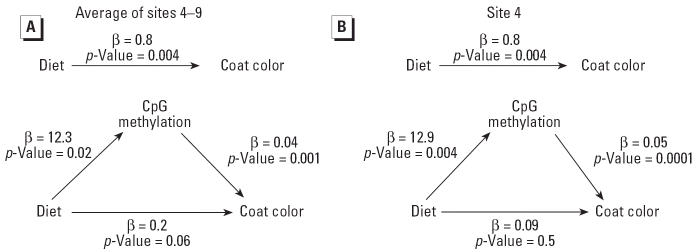
Mediational regression analysis (Baron and Kenny 1986) of genistein diet, *A^vy^* IAP methylation, and coat color. (*A*) CpG methylation variable specified as regional methylation of CpG sites 4–9. Genistein diet significantly influences coat color (top); however, the relationship is reduced when regional *A^vy^* methylation is included in the regression model (bottom). (*B*) CpG methylation variable specified as individual CpG site 4. When CpG site 4 methylation is included in the model (bottom), the direct effect of diet on coat color is abrogated, indicating that methylation at site 4 plays a strong role in mediating the genistein effect on coat color.

The relationship between genistein diet, IAP methylation, and coat color was further assessed by mediational regression analysis (Baron and Kenny 1986) (Figure 3). Genistein diet significantly influences brown coat color (Figure 3A, top), but this relationship was attenuated when regional *A^vy^* CpG methylation of sites 4–9 was included in the model (Figure 3A, bottom). Interestingly, the effect of methylation on coat color was most pronounced when individual site 4 methylation status was specified (Figure 3B), suggesting that site 4 methylation principally mediates the effect of genistein supplementation on *A^vy^*/*a* coat color.

**Figure 4:**
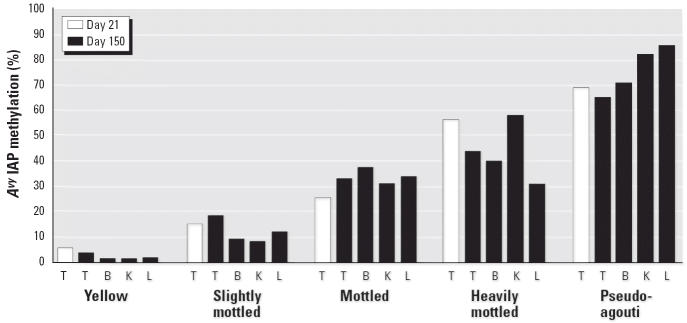
Average *A^vy^* IAP methylation as a function of coat color, tissue type, and age. Abbreviations: B, brain; K, kidney; L, liver; T, tail. Average methylation across CpG sites 1–9 in day 150 tissues derived from ectodermal (B and T), mesodermal (K), and endodermal (L) tissues from five genistein-supplemented *A^vy^*/*a* animals representing the five coat-color phenotypes is correlated with that in day 21 tail tissue (Pearson’s *r* > 0.9 and *p* < 0.05 for each correlation).

Average methylation in day 21 tail tissues from a subset of genistein-supplemented animals (*n* = 5) was highly correlated with average methylation in day 150 tissues derived from the ectoderm (brain and tail), mesoderm (kidney), and endoderm (liver) (Figure 4). Similar results were obtained for individual sites 1–9 (data not shown).

### Adult body weight analysis.

**Figure 5:**
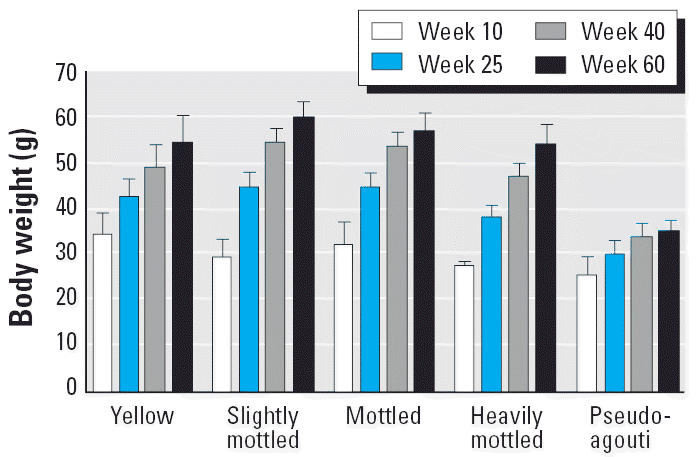
Variation of average body weight among animal coat-color class over time. Significant weight differences among coat-color classes start at week 25 and continue through adulthood. Pseudoagouti animals exhibit normal body weight compared with overweight yellow, slightly mottled, mottled, and heavily mottled animals due to hyper-methylation in the *A^vy^* IAP region, which shuts off ectopic *Agouti* transcription. By shifting the offspring population coat-color distribution toward brown pseudoagouti animals, genistein supplementation significantly increases the incidence of normal-body-weight animals.

Because CpG methylation in the *A^vy^* IAP is inversely correlated to ectopic *Agouti* expression and obesity incidence (Morgan et al. 1999), we determined whether the genistein-induced population shift in coat color also affected body weight distribution in adulthood. First, body weight analysis of all offspring across coat-color classes demonstrated significant differences starting at week 25 and continuing through week 60 (Figure 5). Within coat-color classes, mean week-60 body weights were 54.7 ± 2.8 g for yellow animals (*n* = 9), 59.5 ± 1.7 g for slightly mottled animals (*n* = 24), 56.5 ± 1.9 g for mottled animals (*n* = 15), 54.0 ± 2.0 g for heavily mottled animals (*n* = 14), and 35.6 ± 1.2 g for pseudo-agouti animals (*n* = 12). ANOVA with Bonferroni correction demonstrated that week-60 mean pseudoagouti body weight is significantly reduced when compared with each of the four other coat-color classes (*p* = 0.0001). Significant body weight differences were not observed between the other four coat-color classes.

Second, a higher proportion of genistein-supplemented offspring are classified as pseudoagouti (Figure 2A). Hence, when compared with unsupplemented offspring, genis-tein-supplemented *A^vy^*/*a* offspring at 60 weeks of age were more likely to be of normal weight (< 38 g) and less likely to be obese (> 58 g) (chi-square, *p* = 0.007). Approximately 23% of genistein-supplemented offspring were characterized as normal adult weight, compared with only 10% of unsupplemented offspring. Body weight differences between male and female animals were not observed among all offspring, within diet groups, or within coat-color classes, consistent with sex not being an effect modifier of the relationship between body weight and diet or coat color. When analysis was restricted to animals within the same coat-color class, genistein supplementation was never significantly associated with body weight, indicating that prenatal genistein did not affect body weight via exposure side effects. Thus, the increased methylation resulting from genistein exposure is principally responsible for the population decrease in obesity among genistein offspring.

### SAM and SAH levels.

**Figure 6:**
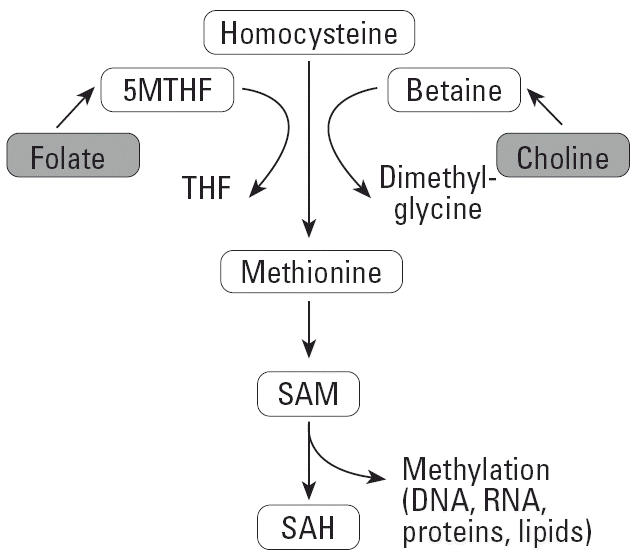
One carbon metabolism pathway. Abbreviations: 5MTHF, 5-methyltetrahydrofolate; THF, tetrahydrofolate. The availability of methyl groups for DNA methylation is increased by provision of excess methyl donors and cofactors, including folate, choline, and SAM, which is the major methyl donor for DNA, RNA, protein, and lipid methylation.

We demonstrated previously that periconceptional supplementation of *A^vy^*/*a* mice with nutrients important to one-carbon metabolism, including folic acid, vitamin B_12_, choline, and betaine, permanently increases offspring average methylation of seven CpG sites in pseudoexon 1A immediately downstream of the *A^vy^* IAP (Waterland and Jirtle 2003). The provision of excess methyl donors and cofactors increases the availability of methyl groups for DNA methylation, as shown in Figure 6. Although genistein is not a methyl donor, the DNA hypermethylation observed in this study could still stem from enhanced efficiency of one or more steps in the one-carbon metabolism pathway. To evaluate this possibility, hepatic concentrations of SAM and SAH were measured in *a*/*a* females that were fed either the unsupplemented or genistein-supplemented diet for 3 weeks. No effect of dietary genistein on SAM or SAH was detected (data not shown).

## Discussion

In the present study, we observed a statistically significant shift in coat-color phenotype and adult body weight distribution among genetically identical offspring whose mothers received a diet supplemented with 250 mg/kg diet of genistein. The shifts in coat color and body weight were mediated by increased methylation at CpG sites 4–6 located immediately upstream of the cryptic promoter region of the *A^vy^* IAP upstream of the transcription start site of the *Agouti* gene. Hypermethylation in the genistein-supplemented population results in decreased ectopic *Agouti* expression, which reduces yellow phaeomelanin production and protects against adult-onset obesity.

In addition to assessing average methylation over the *A^vy^* retrotransposon region, we also determined individual methylation levels at each of the nine CpG sites. The enhanced significance of site 4 coupled with the general increase in methylation closer to the cryptic *A^vy^* promoter suggests that site 4 represents a boundary to methylation spreading and may be particularly important in determining the epigenetically regulated mosaicism in *A^vy^* mouse coat color. Our data indicate that site 4 methylation principally mediates the effect of genistein supplementation on *A^vy^*/*a* coat color. This finding is consistent with methylation status of a single CpG in the glucocorticoid receptor gene promoter principally mediating the effect of maternal caregiving behavior on long-term stress responsiveness in rats (Weaver et al. 2004).

The low variability in CpG methylation among the three germ layer tissues relative to high variability between individual animals indicates that the establishment of epigenotype at the *A^vy^* IAP, which genistein is influencing, occurs early in embryonic development. Furthermore, the concordance between *A^vy^* methylation in day 21 tail and that in the various tissues of the same animal at day 150 demonstrates that genistein-induced epigenetic changes persist to adulthood. The phenomenon of high interindividual coupled with low inter-tissue variability in methylation may represent a common characteristic of epigenetically labile genes in the mouse and human genomes whose expression is controlled by DNA methylation established early in development (Waterland and Jirtle 2004). Consequently, future studies using the *A^vy^* mouse model should more thoroughly investigate the role of stem cells not only in determining cell differentiation early in life but also in promoting cell differentiation during pubertal development.

Body weight data indicate that enhanced IAP methylation in the genistein-supplemented offspring increased the probability that ectopic *Agouti* expression is silenced, leading to a decreased incidence of adult-onset obesity. Using the *A^vy^* mouse model, we have demonstrated for the first time that maternal dietary supplementation is associated with not only altered fetal methylation patterns but also methylation-dependent susceptibility to disease. This finding supports the hypothesis that environmental and nutritional influences on the establishment of epigenetic gene regulatory mechanisms in early life influence adult metabolism and chronic disease susceptibility.

The lack of an association between genis-tein supplementation and SAM or SAH levels indicates that genistein affects DNA methylation through a mechanism that is independent of the one-carbon metabolism pathway. Genistein and other isoflavones interact with the estrogen receptor to enhance histone acetylation (Hong et al. 2004). Therefore, his-tone acetylation may open up the IAP region for methylation, leading to transcriptional deactivation. Whether genistein’s enhancement of DNA methylation is beneficial or deleterious may depend on other environmental factors, such as whether the local food supply is supplemented with folic acid. Because folate is an important cofactor in one-carbon metabolism, individuals who are exposed to folic acid fortification and consume a diet high in soy may experience an additive or even synergistic effect on DNA methylation. Given the recent demonstration of the ability of environmental influences to induce epigenetic changes in the early postnatal period (Weaver et al. 2004), such an interaction could be particularly worrisome for infants fed soy formula diets in which genistein intake relative to body weight reaches levels higher than those used in the present study (Setchell et al. 1997).

The results of our study have a number of other important implications. First, the biologic importance of establishing genomic methylation patterns during early development suggests that it is essential to determine the effects of environmental factors on the epigenome during prenatal and early postnatal development, rather than just in adults. For example, insulin-like growth factor 2 (*IGF2*) loss of imprinting, which places individuals at increased risk of developing colon cancer, is not caused by exposure to adult environmental factors (Cruz-Correa et al. 2004). Rather, it is a trait that is either inherited and/or induced by environmental influences early in embryonic development (Jirtle 2004). Nutritional effects on the fetal epigenome may therefore underlie the long-term cardioprotection of rats born to mothers supplemented with soy during pregnancy (Souzeau et al. 2005). Second, phytoestrogen content in laboratory animal feed is highly variable (Degen et al. 2002). Therefore, genistein’s effect on fetal DNA methylation patterns could significantly influence the interpretation of hormone and other rodent assay studies (Brown and Setchell 2001; Naciff et al. 2004; Wang et al. 2005) as well as confound the interpretation of gene expression arrays and DNA methylation studies. Finally, it needs to be determined whether the relatively high genistein intake of infants consuming soy formulas is beneficial or has unintended deleterious effects on the human epigenome, especially in the United States and other countries where the food supply is fortified with folic acid.

This is the first study to demonstrate that exposure to dietary genistein *in utero*, at levels present in human adult populations consuming high-soy diets, affects coat color and reduces population incidence of obesity by altering the epigenome in mice. Thus, an active ingredient in soy enhances the early establishment of DNA methylation. In addition to single-nucleotide polymorphisms affecting environmentally responsive genes, our findings show that early nutritional and environmentally induced epigenetic modifica-tions can provide an alternative mechanism for varying individual susceptibilities to environmental agents. Our results also suggest a plausible explanation for the lower incidence of certain cancers in Asians compared with Westerners (Chang et al. 2001; Lee et al. 1991) as well as the increased cancer incidence in Asians who immigrate to the United States (Ziegler et al. 1993).

## Correction

In “Materials and Methods,” the authors have clarified that the “modified AIN-93G diet (diet 95092 with 7% corn oil substituted for 7% soybean oil; Harlan Teklad, Madison, WI)” is phytoestrogen-free.
